# Prevalence and Determinants of Depression Among Older Women in Thiruvananthapuram District, Kerala, India: A Community-Based Cross-Sectional Study

**DOI:** 10.7759/cureus.95472

**Published:** 2025-10-26

**Authors:** Lissy Remesan Manju, Navami Sasidharan, Jeby Jose Olickal, Rashita Ravi, P Sankara Sarma, Kavumpurathu R Thankappan

**Affiliations:** 1 Department of Public Health, Amrita Vishwa Vidyapeetham, Kochi, IND; 2 Department of Community Medicine, Amrita Vishwa Vidyapeetham, Kochi, IND

**Keywords:** determinants of health, geriatric mental health, older women, prevalence rate, severe depression

## Abstract

Background

Depression is common among older women and is shaped by various social, economic, and health-related factors. In Kerala, the rapidly aging population and distinct socio-cultural context may influence the prevalence and risk patterns of depression among elderly women. Literature on depression among older women in Kerala is limited. This study assessed the prevalence and determinants of depression among older women in Thiruvananthapuram District, Kerala, India.

Methods

A community-based cross-sectional study was conducted among 480 women aged ≥60 years, selected through multistage cluster sampling. Depression was assessed using the Patient Health Questionnaire (PHQ)-9, and severity was dichotomized as moderately severe or severe (PHQ-9 score ≥15) versus mild or moderate (PHQ-9 score <15). Log-binomial models were used to estimate unadjusted prevalence ratios (UPR), and multivariable Poisson regression was applied to obtain adjusted prevalence ratios (APR) with 95% confidence intervals (CI).

Results

The prevalence of moderately severe or severe depression was 35.8% (n=172, 95% CI: 31.3-40.1). In the multivariable analysis, urban residence (APR=2.83; 95% CI: 2.08-3.86, p<0.001), unmarried status (APR=1.44; 95% CI: 1.15-1.79, p=0.001), presence of chronic morbidity (APR=1.46; 95% CI: 1.10-1.94, p=0.010), and sleep disturbance (APR=1.39; 95% CI: 1.10-1.96, p=0.009) were significantly associated with a higher likelihood of experiencing moderately severe or severe forms of depression.

Conclusion

Over one-third of older women reported moderately severe or severe depression. These findings highlight the need for community-based screening and targeted mental health interventions focusing on urban residents, unmarried individuals, patients with other chronic conditions, and those with sleep disturbances.

## Introduction

Depression affects a significant proportion of the global older adult population, with a pooled prevalence estimated at around 31.7%, and rates being higher in developing countries (40.8%) compared to developed countries (17.0%) [1]. The World Health Organization (WHO) estimates that approximately 14% of adults aged 70 years and above live with a mental disorder, with depression being one of the leading conditions [2]. Globally, women in this age group experience higher rates of depression than men [1]. In India, the prevalence has been reported to be approximately 9.5% among older women, which exceeds the rates observed among older men [3,4]. Kerala, a state in India, with its high proportion of older adults, exhibits even higher prevalence figures among older women, with urban rates approximating 37%, while community-based and rural studies report figures up to 52%, reflecting the influence of diverse social and demographic determinants [5-7]. Institutionalized older women in Kerala also show varied rates of depression, ranging from mild to severe forms [8]. These findings indicate a substantial burden of depression among older women in Kerala, underscoring the need for focused investigations and tailored interventions.

Women aged 60 years and above are particularly vulnerable to depression due to a convergence of biological, social, and economic disadvantages [9,10]. Increased longevity often coincides with the emergence of chronic diseases and physical impairments, heightening susceptibility to mental health challenges [11,12]. Social transitions in later life, such as widowhood, diminished societal roles, and increased financial dependency, contribute to isolation and psychological distress [9,13]. Gender disparities in education, employment, and resource access further compound vulnerability, while psychosocial stressors, including caregiving responsibilities, domestic violence, and cultural expectations, intensify risk [11,13]. Despite the well-recognized burden of geriatric depression, the specific vulnerabilities of older women remain underexplored in actionable detail [2,14].

Although the high prevalence and multifactorial nature of depression in older women are widely acknowledged, research addressing the diverse sociodemographic and regional determinants remains limited [15,16]. Existing studies often generalize across genders or focus on narrow populations, failing to account for the complex interplay of education, family support, socioeconomic status, and living arrangements that uniquely shape women’s mental health in later life [6,8]. Kerala, often regarded as one of India’s most socially empowered states for women, presents a unique context where high literacy rates, better health indicators, and longer life expectancy may influence the risk profile and expression of depression among older women. Therefore, this study aimed to determine the prevalence and factors associated with depression among older women in Thiruvananthapuram district, Kerala.

## Materials and methods

Study design, population, setting, and duration

This community-based cross-sectional study was conducted in Thiruvananthapuram District, Kerala, India, which comprises 78 grama panchayats representing rural areas, one city corporation, and four municipalities representing urban areas, with a total population of approximately 3.3 million according to the 2011 census [17]. The study population included women aged 60 years and above who were permanent residents of the selected study area (those who had continuously lived in the area for at least one year prior to the survey and considered it their usual place of residence). Data collection was carried out between December 2024 and February 2025. The study protocol was reviewed and approved by the Ethics Committee of Amrita School of Medicine, Kochi (Reference number: ECASM-AIMS-2024-599).

Inclusion and exclusion criteria

Women aged 60 years or older who were permanent residents of the study area, irrespective of their marital status, employment status, or educational level, and who provided informed consent and were available during the survey period were included in the study.

Women who were critically ill, bedridden, had significant cognitive impairment, or were unable to communicate effectively during the interview were excluded.

Sample size and sampling technique

The sample size was calculated based on a reported prevalence of depression of 24.02% from a community-based study in Kerala [18]. With 5% absolute precision, 95% confidence level, and a design effect of 1.5, the required sample size was 423. After adjusting for a 10% non-response rate, the sample size was increased to 470 and rounded to 480 to ensure equal recruitment across 24 clusters (20 participants per cluster).

A multistage cluster sampling technique was adopted. In the first stage, 12 rural wards (a ward is the smallest administrative unit in Kerala) were selected from two grama panchayats (six wards each), and 12 urban wards were selected, with six chosen from the corporation and six from the selected municipality. The selection of wards was done using simple random sampling by the lottery method to ensure equal probability of inclusion. In the second stage, 20 households were selected in each chosen ward. The first household was identified from a random starting point within the ward, and continuous sampling was carried out along one side of the street until the required number of 20 households was reached. From each selected household, one eligible woman aged 60 years or above was recruited. If an eligible participant was unavailable or unwilling, the next adjacent household was immediately approached as a replacement.

Data collection

Data were collected through one-to-one interviews using a pre-tested semi-structured questionnaire that was translated into Malayalam, the local language. The translated version was pre-tested among 10 participants who were not included in the main study, and minor wording adjustments were made to improve clarity. The questionnaire comprised two sections for depression assessment: (1) Socio-demographic and health-related characteristics, and (2) Patient Health Questionnaire-9 (PHQ-9) [19].

Study variables

Independent variables included age, education, place of residence, socioeconomic status, religion, marital status, pension status, social participation, presence of chronic morbidity, and sleep disturbance. Socioeconomic status was classified as Above Poverty Line (APL) or Below Poverty Line (BPL), based on the type of government-issued ration card possessed by the household. The variable social participation was included as part of the socio-demographic information obtained through the semi-structured questionnaire, rather than being measured using a separate standardized tool.

The outcome variable was depression, classified according to PHQ-9 score categories as follows [19]: (1) <5: No depression; (2) 5-9: Minimal to mild depression; (3) 10-14: Moderate depression; (4) 15-19: Moderately severe depression; (5) 20-27: Severe depression.

The detailed questionnaire is shown in Table 1.

**Table 1:** Questionnaire COPD: Chronic Obstructive Pulmonary Disease; PHQ: Patient Health Questionnaire [19]; Socioeconomic status was classified as Above Poverty Line (APL) or Below Poverty Line (BPL), based on the type of government-issued ration card possessed by the household.

Sl. no	Question	Responses
Sociodemographic and health realted characteristics		
1	Age (in completed years)	
2	Education level	Up to middle school/High school and above
3	Place of residence	Urban/Rural
4	Socioeconomic status	Above the poverty line (APL)/Below the poverty line (BPL)
5	Religion	Hindu/Muslim/Christian/Other (specify)
6	Marital status	Currently married/Divorced/Separated/Widowed/Unmarried
7	Do you receive any pension?	Yes/No
8	Do you participate in any social or community activities?	Yes/No
9	Do you have any chronic morbidity? (Diabetes, hypertension, chronic heart disease, asthma, COPD, or mental health problems)	Yes/No
10	Do you experience sleep disturbances?	Yes/No
PHQ-9 Item*		Responses
11	Little interest or pleasure in doing things	Not at all/Several days/More than half the days/Nearly every day
12	Feeling down, depressed, or hopeless	Not at all/Several days/More than half the days/Nearly every day
13	Trouble falling or staying asleep, or sleeping too much	Not at all/Several days/More than half the days/Nearly every day
14	Feeling tired or having little energy	Not at all/Several days/More than half the days/Nearly every day
15	Poor appetite or overeating	Not at all/Several days/More than half the days/Nearly every day
16	Feeling bad about yourself — or that you are a failure or have let yourself or your family down	Not at all/Several days/More than half the days/Nearly every day
17	Trouble concentrating on things, such as reading the newspaper or watching television	Not at all/Several days/More than half the days/Nearly every day
18	Moving or speaking so slowly that other people could have noticed? Or the opposite — being so fidgety or restless that you have been moving around a lot more than usual	Not at all/Several days/More than half the days/Nearly every day
19	Thoughts that you would be better off dead, or of hurting yourself in some way	Not at all/Several days/More than half the days/Nearly every day

Statistical analysis

Data were entered using EpiCollect5 (Centre for Genomic Pathogen Surveillance. (2025). Epicollect5. Available at: https://five.epicollect.net. Last accessed: October 26, 2025) and analyzed with STATA version 14 (StataCorp LLC, College Station, TX, US). Categorical variables were summarized as frequencies and percentages. The prevalence of depression was calculated with 95% confidence intervals (CI). For bivariate and multivariable analyses, severity of depression was dichotomized into moderately severe or severe depression (PHQ-9 score ≥15) and mild or moderate depression (PHQ-9 score <15). The association between sociodemographic factors with moderately severe or severe depression was studied using bivariate Poisson regression, and unadjusted prevalence ratios (UPR) were estimated. Variables with p values<0.2 in the bivariate analysis were entered into a multivariable Poisson regression model with robust variance to estimate adjusted prevalence ratios (APR) and 95% CI. A p-value<0.05 was considered statistically significant. 

## Results

Socio-demographic and health-related characteristics of the study participants are presented in Table 2.

**Table 2:** Socio-demographic and health related factors among study participants (N=480) ^*^Others include divorced, separated, widowed, or unmarried participants; ^#^Diabetes, hypertension, chronic heart disease, asthma, chronic obstructive pulmonary diseases, or known mental health issues

Variables	Category	Frequency (n)	Percentage (%)
Age categories	<70 years	244	50.8
	≥70 years	236	49.2
Education level	Up to middle school	146	30.4
	High school and above	334	69.6
Place of residence	Urban	240	50.0
	Rural	240	50.0
Socioeconomic status (based on ration card)	Above the poverty line	310	64.6
	Below the poverty line	170	35.4
Religion	Hindu	429	89.4
	Others (Muslim / Christian)	51	10.6
Marital status	Currently married	255	53.1
	Others^*^	225	46.9
Receiving pension	Yes	385	80.2
	No	95	19.8
Social participation	Yes	83	17.3
	No	397	82.7
Any chronic morbidity^#^	Yes	308	64.2
	No	172	35.8
Sleep disturbances	Yes	308	64.2
	No	172	35.8

Age was recorded as a continuous variable and later categorized for analysis. The participants’ ages ranged from 60 to 109 years, with nearly half (49.2%) aged 70 years or above. The majority had attained high school education or above (n=334; 69.6%), and nearly two-thirds belonged to households above the poverty line (n=310; 64.6%). Most participants identified as Hindu (429; 89.4%). Slightly more than half were currently married (n=255; 53.1%), the majority were receiving a pension (n=385; 80.2%), and most reported no social participation (n=397; 82.7%). Chronic morbidity and sleep disturbances were each reported by 308 participants (64.2%).

The prevalence of depression is summarized in Table 3.

**Table 3:** Prevalence of depression among study participants (N=480) CI: confidence interval; PHQ: Patient Health Questionnaire.

PHQ-9 Category	Frequency (n)	Percentage (%)	95% CI
Mild depression	5	1.0	0.3-2.4
Moderate depression	303	63.1	58.6-67.4
Moderately severe depression	171	35.6	31.3-40.1
Severe depression	1	0.2	0.0-1.1

Mild depression was reported by five participants (1.01%; 95% CI: 0.31-2.44), moderate depression by 303 (63.12%; 95% CI: 58.62-67.44), moderately severe depression by 171 (35.64%; 95% CI: 31.32-40.11), and severe depression by one participant (0.23%; 95% CI: 0.01-1.10).

Table 4 presents the factors associated with moderately severe or severe depression.

**Table 4:** Factors associated with moderately severe or severe depression among study participants, results from bivariate and multivariable models (N=480) APL: Above the poverty line; BPL: Below the poverty line; CI: Confidence interval; UPR: Unadjusted Prevalence Ratio; APR: Adjusted Prevalence Ratio. Reference categories are indicated as 1.

Variables	Moderately severe or severe depression n (%)	Mild or moderate depression n (%)	UPR (95% CI)	P-value	APR (95% CI)	P-value
Age (years)						
<70	88 (36.1)	156 (63.9)	1.01 (0.80–1.29)	0.914	—	—
≥70	84 (35.6)	152 (64.4)	1	—	—	—
Education level						
High school and above	132 (39.5)	202 (60.5)	1.44 (1.07–1.94)	0.015	1.21 (0.90–1.63)	0.195
Middle school	40 (27.4)	106 (72.6)	1	—	1	—
Place of residence						
Urban	131 (54.6)	109 (45.4)	3.20 (2.36–4.32)	<0.001	2.83 (2,–3.86)	<0.001
Rural	41 (17.1)	199 (82.9)	1	—	1	
Socioeconomic status (based on ration card)						
APL	124 (40.0)	186 (60.0)	1.42 (1.08–1.87)	0.013	1.09 (0.84–1.42)	0.492
BPL	48 (28.2)	122 (71.8)	1	—	1	—
Religion						
Hindu	162 (37.8)	267 (62.2)	1.93 (1.09–3.40)	0.024	1.35 (0.79–2.34)	0.268
Others	10 (19.6)	41 (80.4)	1	—	1	—
Marital status						
Others	103 (45.8)	122 (54.2)	1.69 (1.32–2.17)	<0.001	1.44 (1.15–1.79)	0.001
Currently married	69 (27.1)	186 (72.9)	1	—	1	—
Receiving pension						
No	37 (38.9)	58 (61.1)	1.11 (0.83–1.48)	0.472	—	—
Yes	135 (35.1)	250 (64.9)	1	—	—	—
Any chronic morbidity						
Yes	130 (42.2)	178 (57.8)	1.73 (1.29–2.32)	<0.001	1.46 (1.10–1.94)	0.010
No	42 (24.4)	130 (75.6)	1	—	1	—
Sleep disturbance						
Yes	118 (38.3)	190 (61.7)	1.22 (0.94–1.59)	0.137	1.37 (1.10–1.96)	0.007
No	54 (31.4)	118 (68.6)	1	—	1	—

Urban residence was significantly associated with a higher likelihood of moderately severe or severe depression compared to rural residence (APR=2.83; 95% CI: 2.08-3.86; p<0.001). Participants who were divorced, separated, widowed, or unmarried were more likely to have moderately severe or severe depression compared to those who were currently married (APR=1.44; 95% CI: 1.15-1.79; p=0.001). The presence of any chronic morbidity (APR=1.46; 95% CI: 1.10-1.94; p=0.010) and sleep disturbance (APR=1.39; 95% CI: 1.10-1.96; p=0.009) were also significantly associated with moderately severe or severe depression.

## Discussion

Our study among older women in Thiruvananthapuram district reveals a high burden of depression, with 63.1% reporting moderate depression and 35.8% reporting moderately severe or severe depression, figures that lie at the upper end of the wide range reported from Kerala and India. Urban community studies from southern India have documented depression comparable to our estimates, while several rural Kerala studies have also reported substantial prevalence, underscoring contextual heterogeneity [16,18]. Pooled Indian estimates typically range between 30% and 40%, placing our estimates above national averages in this subgroup of older women [4,20]. Globally, a systematic review among older adults reports markedly lower point prevalence for depression; however, variations in screening tools, cut-off thresholds, and distinctions between symptom scales and diagnostic criteria complicate direct comparisons [21]. Overall, these findings support existing evidence from Indian and international reviews that later-life depression is common yet context-dependent, and that women, particularly in settings undergoing rapid social change, experience a disproportionate burden of depression [10,13]. 

Urban residence independently predicted a higher likelihood of moderately severe or severe depression, consistent with findings from Kerala and national Indian studies linking city living to mental health disadvantages via factors such as crowding, cost-of-living stress, weaker kinship networks, and environmental stressors [5,10]. However, high prevalence rates reported from rural Kozhikode District of Kerala and mixed rural cohorts indicate that urban-rural differences likely reflect varying risk profiles rather than a simple linear gradient [7,16]. Socioeconomic conditions, living arrangements, and access to care may shape these patterns, explaining why some rural populations appear equally or more vulnerable [10]. In Thiruvananthapuram district, urban environments may contribute additional psychosocial stress and caregiving burdens that intensify depression, without contradicting the evidence of substantial rural burden elsewhere in Kerala [18,22].

Being divorced, separated, widowed, or unmarried was strongly associated with a higher prevalence of moderately severe or severe depression compared with being currently married, aligning with multi-country and Indian studies identifying spousal absence as a major determinant of late-life depression through pathways such as bereavement, loneliness, loss of emotional and financial support, and income insecurity [12,13]. Kerala-based studies have similarly reported elevated depressive symptoms among women lacking spousal support, reflecting longer female life expectancy and traditional caregiving roles that often leave them isolated in later life [6,18]. The protective effect of cohesive family ties and social participation may be weakened when marital support is absent, exacerbating psychological distress [10,12].

Presence of any other chronic morbidity also emerged as an independent predictor, with women having chronic conditions showing a 46% higher adjusted prevalence of moderately severe or severe depression. This finding is consistent with evidence from rural Odisha and other Indian meta-analyses, which also reported higher depression rates among individuals with chronic diseases such as diabetes, cardiovascular diseases, and respiratory illnesses, likely due to systemic inflammation, symptom burden, and functional limitations [10,11]. International reviews consistently identify physical illness and disability as strong determinants of late-life depression, with bidirectional effects where depression worsens treatment adherence and disease control [9,12]. These findings highlight the need for integrated care models in Kerala by embedding PHQ-9 screening within non-communicable disease (NCD) clinics, offering spousal survivor support, grief counseling, and social engagement programs to reduce depression risk and enhance well-being among older women [9-13,18].

Sleep disturbance was independently associated with moderately severe or severe depression, consistent with extensive evidence linking sleep and mood in a bidirectional cycle, where insomnia and fragmented sleep worsen emotional regulation and inflammatory processes, while depression further impairs sleep quality and continuity. Reviews on late-life depression consistently highlight poor sleep as both a correlate and a modifiable target for intervention, with several Indian community studies reporting similar associations [9,12]. Among older women, menopausal changes, chronic pain, and night time caregiving responsibilities may aggravate sleep problems, making insomnia an important entry point for low-cost, scalable interventions such as sleep hygiene education, brief behavioral therapies, and, when necessary, pharmacological support. Our findings emphasize the importance of routinely assessing sleep in primary care visits and implementing community-based interventions that can be delivered by frontline health workers within Kerala’s mixed urban-rural health system [10,12].

This study has certain limitations. The cross-sectional design precludes establishing temporal or causal relationships between associated factors and moderately severe or severe depression. The use of self-reported data for variables such as social participation, chronic morbidity, and sleep disturbance may introduce recall or reporting bias. Although the PHQ-9 is a widely validated screening tool, it does not substitute for a clinical diagnosis, and some misclassification may have occurred. The study was restricted to older women in Thiruvananthapuram district, which may limit generalizability to men, other districts, or different sociocultural contexts. Additionally, residual confounding from unmeasured factors, such as past psychiatric history, quality of family support, and life events, may have influenced the observed associations.

## Conclusions

This study revealed that moderately severe or severe depression was observed in more than one-third of the older women assessed in Thiruvananthapuram district. Depression was more common among those living in urban areas, women who were divorced, separated, widowed, or unmarried, and those with chronic morbidities or sleep disturbances. These findings indicate that both social and health-related factors play a crucial role in late-life mental health. Regular mental health screening should be incorporated into routine geriatric care, with age- and gender-sensitive services integrated into existing programs such as the National Programme for Health Care of the Elderly (NPHCE) and the National Mental Health Programme (NMHP). Addressing late-life depression requires a coordinated strategy combining healthcare integration, caregiver education, and community engagement to promote mental well-being and dignity among older women in Kerala and similar settings.
